# Gene expression profiling of epithelial ovarian tumours correlated with malignant potential

**DOI:** 10.1186/1476-4598-3-27

**Published:** 2004-10-07

**Authors:** Susanne Warrenfeltz, Stephen Pavlik, Susmita Datta, Eileen T Kraemer, Benedict Benigno, John F McDonald

**Affiliations:** 1 Genetics Department, University of Georgia, Athens Georgia 30602, USA; 2 Ovarian Cancer Institute, Atlanta Georgia, 30342, USA; 3 Computer Science, University of Georgia, Athens, Georgia 30602, USA; 4 Mathematics and Statistics, Georgia State University, Atlanta, Georgia 30303, USA

## Abstract

**Background:**

Epithelial ovarian tumours exhibit a range of malignant potential, presenting distinct clinical phenotypes. Improved knowledge of gene expression changes and functional pathways associated with these clinical phenotypes may lead to new treatment targets, markers for early detection and a better understanding of disease progression.

**Results:**

Gene expression profiling (Affymetrix, U95Av2) was carried out on 18 ovarian tumours including benign adenomas, borderline adenocarcinomas of low malignant potential and malignant adenocarcinomas. Clustering the expression profiles of samples from patients not treated with chemotherapy prior to surgery effectively classified 92% of samples into their proper histopathological group. Some cancer samples from patients treated with chemotherapy prior to surgery clustered with the benign adenomas. Chemotherapy patients whose tumours exhibited benign-like expression patterns remained disease free for the duration of this study as indicated by continued normal serum CA-125 levels. Statistical analysis identified 163 differentially expressed genes: 61 genes under-expressed in cancer and 102 genes over-expressed in cancer. Profiling the functional categories of co-ordinately expressed genes within this list revealed significant correlation between increased malignant potential and loss of both IGF binding proteins and cell adhesion molecules. Interestingly, in several instances co-ordinately expressed genes sharing biological function also shared chromosomal location.

**Conclusion:**

Our findings indicate that gene expression profiling can reliably distinguish between benign and malignant ovarian tumours. Expression profiles of samples from patients pre-treated with chemotherapy may be useful in predicting disease free survival and the likelihood of recurrence. Loss of expression of IGF binding proteins as well as specific cell adhesion molecules may be a significant mechanism of disease progression in ovarian cancer. Expression levels in borderline tumours were intermediate between benign adenomas and malignant adenocarcinomas for a significant portion of the differentially expressed genes, suggesting that borderline tumours are a transitional state between benign and malignant tumours. Finally, genes displaying coordinated changes in gene expression were often genetically linked, suggesting that changes in expression for these genes are the consequence of regional duplications, deletions or epigenetic events.

## Background

Epithelial ovarian cancer is the fifth leading cause of death for women in the United States [1]. Although early stage ovarian cancer can be effectively treated, symptoms of early disease are sufficiently vague that accurate diagnosis is often delayed until the cancer has progressed into more advanced stages [2]. Treatment of early staged tumours (I through IIa) is associated with a 5-year survival rate of approximately 95% while survival rates drop to less than 30% when diagnosis is delayed until later stages (stage IIb through IV). To improve these statistics, effective early diagnosis and treatment strategies must be developed. Further knowledge of the genes and gene functional pathways involved in ovarian cancer are needed in order to develop these strategies.

Microarray technology has revolutionised the study of gene function by providing "snapshots" of global gene expression patterns from different normal and diseased tissues over multiple stages of development. Nowhere has the impact of this technology been more pronounced than in the field of cancer biology where gene expression profiling has been successfully used to objectively classify tumours and, in some instances, identify novel tumour sub-types [3]. Microarray analyses have also been instrumental in the elucidation of new biological pathways that may be involved in tumour development, as well as, in the identification of new biomarkers of the disease and potential targets of therapeutic intervention.

Previous microarray studies of ovarian cancers have focused on the characterisation of differences between normal ovarian epithelial cells (and cell lines) and various types and stages of ovarian tumours [4-10]. In this study, we focus on characterising differences between benign adenomas, borderline tumours of low malignant potential and malignant adenocarcinomas in order to identify changes associated with the acquisition of malignancy and to avoid the technical difficulties associated with obtaining sufficient amounts of normal ovarian surface epithelium. The ovarian tumour tissue samples used in these microarray studies were chosen to accurately represent the range of malignant potential observed clinically.

We report here the results of applying clustering and statistical analyses to the microarray expression profiles of 18 ovarian tumours. Our findings indicate that gene expression profiling distinguished properly classify 92% of tumours in this study as benign or malignant. Samples taken from ovarian cancer patients who had been treated with chemotherapy prior to surgery were found not to cluster as a distinct group but rather with either the benign or malignant (not pre-treated) tumours. Chemotherapy patients whose tumours clustered with the benign group remained disease free for the duration of the study as evidenced by continued normal serum CA-125 levels. Profiling the functional categories of co-ordinately expressed genes revealed significant correlation between increased malignant potential and loss of IGF binding proteins, and cell adhesion molecules. In several instances co-ordinately expressed genes sharing functional categories also correlated with chromosomal location.

## Results

### Unsupervised clustering of gene expression profiles can reliably identify ovarian tumour types

**Figure 1:**
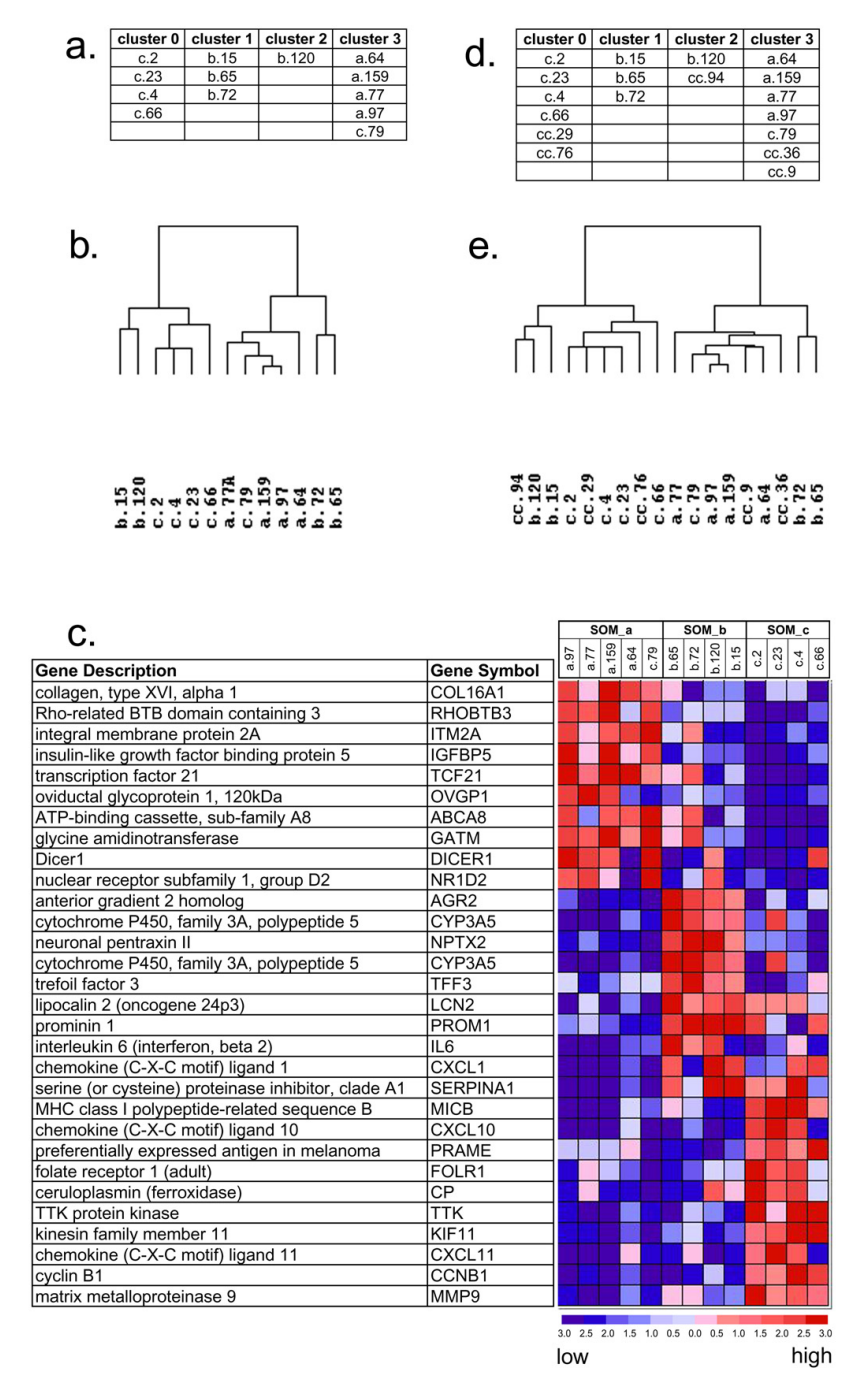
**Cluster analysis of ovarian tumour expression profiles. **Gene expression profiles were obtained from eighteen ovarian tumours. The profiles were analysed by clustering methods in several groups: (a) self organizing map of he thirteen patients not receiving chemotherapy prior to tissue collection, (b) hierarchical clustering of the same 13 patients, (d) self organizing maps of all eighteen patients and (e) hierarchical clustering of all 18 patients. Marker analysis (c) identified the top ten gene most highly correlated with clusters resulting from (a) and (b).

To determine if gene expression profiling can distinguish between histologically determined tumour types, we analysed the profiles of 13 ovarian tumours (Table 1) by performing clustering using self-organising maps (SOM) and unsupervised hierarchical clustering (UHC). Self-organising maps are a type of mathematical cluster analysis used to recognise and classify features in complex multi-dimensional data [11]. SOMs group samples into a user-defined number of clusters based on the similarity of the gene expression profiles. The set of thirteen samples was comprised of four benign adenomas (a_64, a_77, a_97, a_159), four borderline tumours of low malignant potential (b_15, b_65, b_72, b_120) and five malignant adenocarcinomas (c_2, c_4, c_23, c_66, c_79). Analysing all 12,590 probe set values from the 13 samples into four groups resulted in 92% of the samples being grouped into clusters consistent with their histopathological classification (Figure 1a). One cluster (cluster 0) contained only adenocarcinomas, two clusters (clusters 1 and 2) contained only borderline tumours, and one cluster (cluster 3) contained all of the benign adenomas and one adenocarcinoma sample. In addition, the UHC of the entire data set (Figure 1b) produced essentially the same clusters as determined by SOM. The only difference between the SOM and UHC results was the stratification of borderline tumours, which are known to be a heterogeneous group of tumours. The SOM clustered the four borderline samples into one group of three borderlines (b_65, b_15, b_72) and one solitary sample (b_120) (Figure 1a). However, the UHC clustered the four borderline samples into one group containing b_15 and b_120, and one group containing b_65, and b_72. Since c_79 was consistently misclassified, a second tissue sample of c_79 was analysed by microarray and clustered as above. This independently obtained expression profile for c_79 produced the same results.

**Table 1:** Tissue Sample Information

Tumor ID	Malignant Potential	Available Histological Information	Stage	Chemo
a_64	benign	Serous cystadenofibroma	-	-
a_77	benign	Serous cystadenofibroma	-	-
a_97	benign	Serous cystadenoma	-	-
a_159	benign	Serous cystadenofibroma	-	-
b_15	low/borderline	Serous papillary adenocarcinoma	III	-
b_65	low/borderline	Mucinous adenocarcinoma	II	-
b_72	low/borderline	Mucinous adenocarcinoma	I	-
b_120	low/borderline	Serous papillary adenocarcinoma	II	-
c_2	invasive malignant	Serous papillary adenocarcinoma	IIb	-
c_4	invasive malignant	Serous papillary adenocarcinoma	III	-
c_23	invasive malignant	Serous papillary adenocarcinoma	IIIa	-
c_66	invasive malignant	Serous papillary/endometroid adenocarcinoma	IV	-
c_79	invasive malignant	Serous papillary carcinoma	III	-
cc_9	invasive malignant	Serous papillary carcinoma	III	Yes*
cc_29	invasive malignant	Serous papillary carcinoma	III	Yes^#^
cc_36	invasive malignant	Serous papillary adenocarcinoma	IIIc	Yes*
cc_76	invasive malignant	Serous papillary adenocarcinoma	IIIa	Yes*
cc_94	invasive malignant	Serous carcinoma	III	Yes*

* Taxol/Carbo 3X prior to surgery #Taxol/Carbo 4X prior to surgery

Since many of the genes in our data set display no differential expression across the 13 tumours, their contribution to the SOM is negligible and can be considered noise. Removing genes whose expression pattern displayed insignificant variation (low standard deviation) across all samples, we reduced the data set to1000 probe sets. After removing probe sets representing the same gene, the reduced data set contained expression values representing 700 genes. The SOM and UHC of the reduced data set yielded identical clusters to those obtained using the entire data set (Figure 1a and 1b).

To determine the genes most highly correlated with each cluster identified by the SOM analysis, we performed a marker analysis (Figure 1c) on the reduced set of 700 genes. Marker analysis helps the user discover which genes are most closely correlated with a cluster and provides a measure of how significant that correlation is for each gene. Marker analysis measures the contribution of each gene to the SOM groupings based on a signal to noise ratio calculated from the difference in each gene's mean expression scaled by the sum of the standard deviations across all samples. To avoid having one cluster containing only one sample in the marker analysis, we grouped clusters cluster 1 and cluster 2 containing the borderline samples together, creating three clusters (Figure 1c) consisting of the benign adenomas and c79 (SOM_a), borderline adenocarcinomas (SOM_b), and the malignant adenocarcinomas (SOM_c). Genes highly correlated with each SOM group were expressed strongly in the tumour type associated with that SOM group and poorly expressed in the other SOM groups. It is interesting to note that the 10 genes highly correlated with SOM_a were expressed at intermediate levels in borderline tumours. Similarly, the 10 genes highly correlated with SOM_b were expressed at intermediate levels in the adenocarcinomas of SOM_c.

### Gene expression profiles are correlated with recurrence

Five of the cancer patients in our study were treated with chemotherapy prior to surgery. We added the microarray profiles of these patient samples to our analysis in order to determine if they would cluster into a new distinct group or into one or more of the existing groups. The SOM (Figure 1d) and UHC (Figure 1e) clusters resulting from the analysis of all data (12,590 expression values) from all eighteen samples into four clusters differ only in the stratification of the borderline samples. The addition of the five samples from patients who received chemotherapy prior to surgery did not change the cluster assignments of the original thirteen samples. Clustering of the reduced set of 700 genes (see above), resulted in the same patterns of clustering as determined using the entire set of 12,590 expression values (Figure 1d and 1e). Interestingly, the five samples from patients pre-treated with chemotherapy did not cluster together in a distinct group but rather were dispersed among the existing four clusters. Samples cc_29 and cc_76 clustered with the malignant adenocarcinomas, while samples cc_36 and cc_9 clustered with the benign adenomas. Sample cc_94 clustered with the borderline tumours.

**Figure 2:**
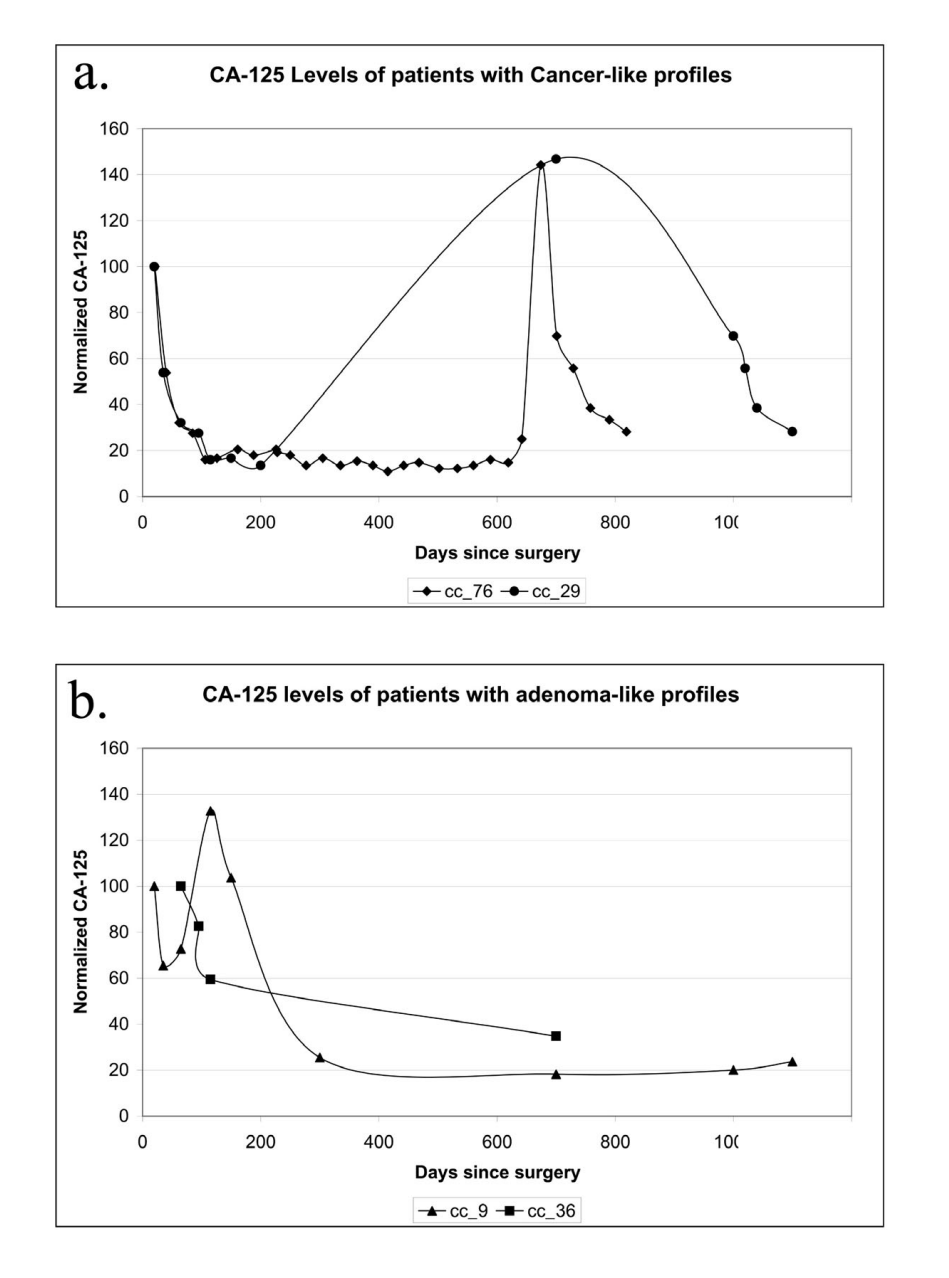
**CA-125 levels of patients receiving chemotherapy prior to tissue collection. **CA-125 levels of patients with cancer-like profiles (a) and adenoma-like profiles (b) were normalized to the earliest post-surgery reading. CA-125 level for patients 76 and 29, two patients receiving chemotherapy prior to tissue collection, spike dramatically at about 700 days post surgery. CA-125 levels for patients 9 and 36 remained low through 700 days past surgery.

In an initial effort to test the possible clinical significance of the differential clustering of samples obtained from patients pre-treated with chemotherapy, we examined the post-operative history of these patients. One commonly used indicator of recurrence is the level of Cancer Antigen-125 (CA-125) in the blood [12,13]. Although post-operative CA-125 levels were initially lowered to a significant extent in all of the patients pre-treated with chemotherapy, the levels remained consistently low in only those patients (cc_36, cc_9) whose microarray profiles clustered with the benign adenomas (Figure 2). The remaining patients displayed periodic recurrence requiring additional chemotherapy.

### Significant differences in gene expression are associated with different ovarian tumour types

To identify genes whose differential expression correlate with malignant potential, we performed a statistical analysis comparing the expression profiles of the three tumour types examined in this study (benign adenoma, low malignant potential borderline adenocarcinoma, and malignant adenocarcinoma). Malignant adenocarcinoma sample c_79 was excluded from this analysis since both the SOM and UHC classification methods identified this sample as an outlier of the malignant adenocarcinoma group (see above). The F statistic was used to test equality of group means [14]. Genes whose group means were identified as significantly different (p ≤ 0.001, 299 genes) in the ANOVA analysis were further analyzed using multiple comparison methods to determine which means differ from each other. The differences between group means for all pairwise combinations of groups were calculated and compared to the least significant difference. Genes were declared differentially expressed if the pairwise difference between group means was greater than the least significant difference. Probe sets duplicated between pairwise comparisons and probes sets with a fold change value below 2.0 were removed, leaving 163 unique genes differentially expressed between the tumour groups. The 15 differentially expressed genes with highest statistical significance are presented in Table 2. The gene name, gene symbol, chromosomal location, functional classification, ANOVA rank and p-value of each of these 163 genes are attached as additional file 1 (complete list.txt).

**Table 2:** Highest 15 statistically significant genes via ANOVA analysis, their fold change and p-values.

**Affy ID**	**Gene Name**	**Gene Symbol**	**FC a:b**	**FC a:c**	**FC c:b**	**ANOVA p-value**
1651_at	ubiquitin-conjugating enzyme E2C	UBE2C	1.16(b)	4.35(c)	3.75(c)	1.2E-07
41583_at	flap structure-specific endonuclease 1	FEN1	1.27(b)	3.86(c)	3.04(c)	1.9E-07
31888_s_at	tumour suppressing subtransferable candidate 3	TSSC3	3.46(b)	8.15(c)	2.36(c)	2.7E-07
34715_at	forkhead box M1	FOXM1	1.06(b)	2.66(c)	2.5(c)	7.5E-07
39109_at	chromosome 20 open reading frame 1	C20orf1	1.29(b)	4.91(c)	3.80(c)	2.9E-06
37985_at	lamin B1	LMNB1	1.19(b)	3.19(c)	2.69(c)	2.9E-06
41451_s_at	SAR1 protein	SAR1	1.07(b)	2.27(c)	2.13(c)	3.2E-06
37015_at	aldehyde dehydrogenase 1 family, member A1	ALDH1A1	1.86(a)	10.76(a)	5.79(b)	3.3E-06
527_at	centromere protein A, 17kDa	CENPA	1.08(b)	3.47(c)	3.2(c)	3.9E-06
40619_at	ubiquitin carrier protein	E2-EPF	1.51(b)	2.74(c)	1.81(c)	4.6E-06
32332_at	isocitrate dehydrogenase 2 (NADP+), mitochondrial	IDH2	1.28(b)	4.36(c)	3.40(c)	5.4E-06
1058_at	WAS protein family, member 3	WASF3	2.03(a)	2.56(a)	1.27(b)	5.7E-06
1943_at	cyclin A2	CCNA2	1.05(a)	2.07(c)	2.17(c)	6.4E-06
2039_s_at	FYN oncogene related to SRC, FGR, YES	FYN	1.05(b)	3.11(a)	2.97(b)	6.4E-06
1868_g_at	CASP8 and FADD-like apoptosis regulator	CFLAR	0.97(b)	1.78(c)	1.83(c)	7.7E-06

**Figure 3:**
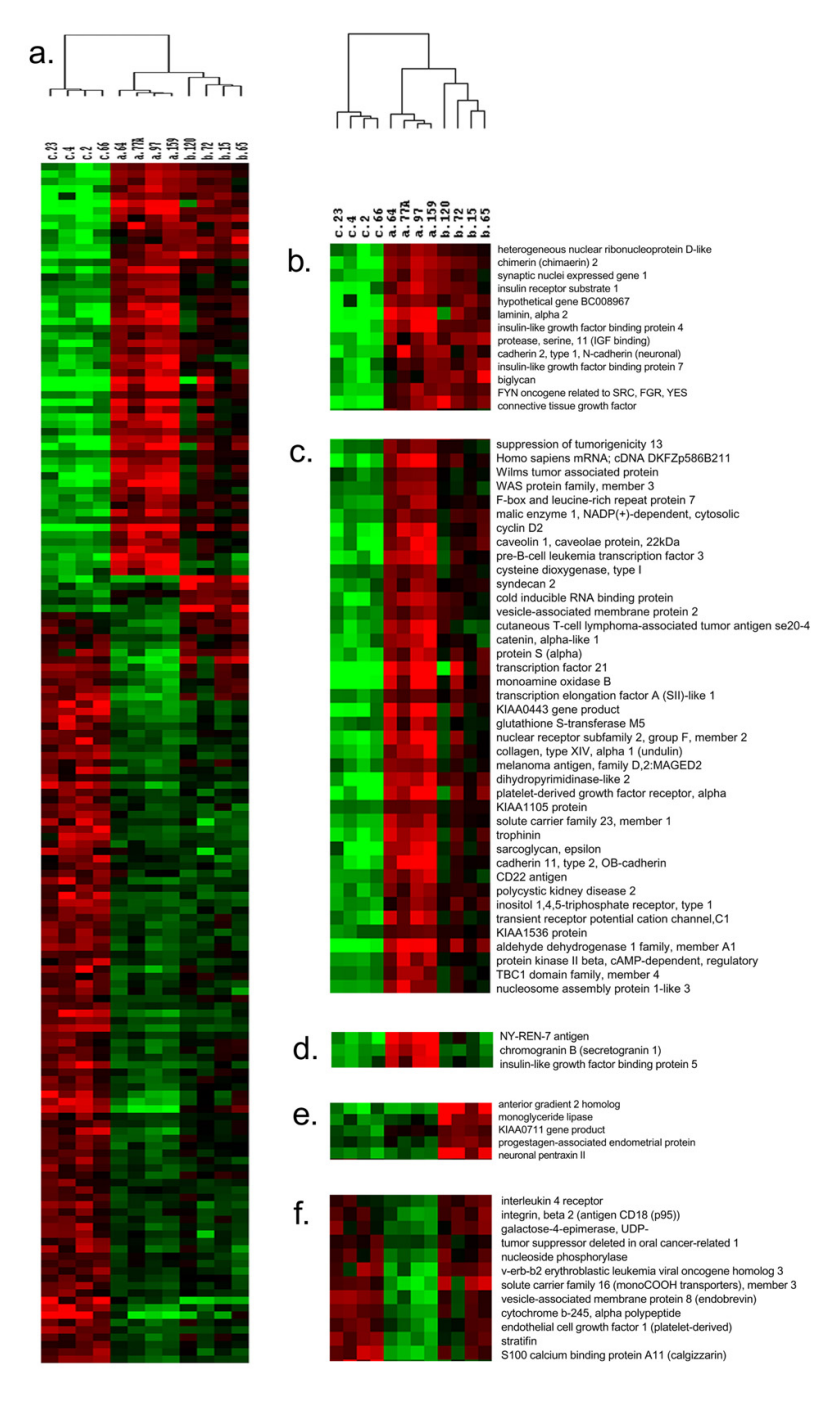
**Patterns of differential expression for the 163 genes of highest statistical significance. **The 300 probe sets with the lowest p-values in the ANOVA analysis were filtered for duplicate genes and fold change <2.0. The remaining 163 genes were subjected to hierarchical clustering to reveal correlated expression (a). Thirteen genes showed high expression in both benign adenomas and borderline tumours (b). Borderline tumours showed intermediate levels of expression for forty genes (c). Three genes were high only in benign adenoma (d). Five genes showed high expression in borderline tumours only (e). And 13 genes were high in both malignant adenocarcinomas and borderline tumours (f).

Hierarchical clustering was performed to visualise gene expression across tumour types for each of these 163 genes. All 12 tumours were correctly assigned as shown by the dendogram above the gene expression colour plot (Figure 3a). Several features within the gene expression colour plot are worthy of note (Figure 3b,3c,3d,3e,3f). Thirteen genes (Figure 3b) showed high expression in both adenoma and borderline. For forty genes expression levels in borderline tumours was intermediate between adenoma and cancer (Figure 3c). Eight genes were highly expressed in either adenoma (3 genes, Figure 3c) or borderline (5 genes, Figure 3d). And finally, thirteen genes showed high expression in both cancer and borderline (Figure 3e).

**Figure 4:**
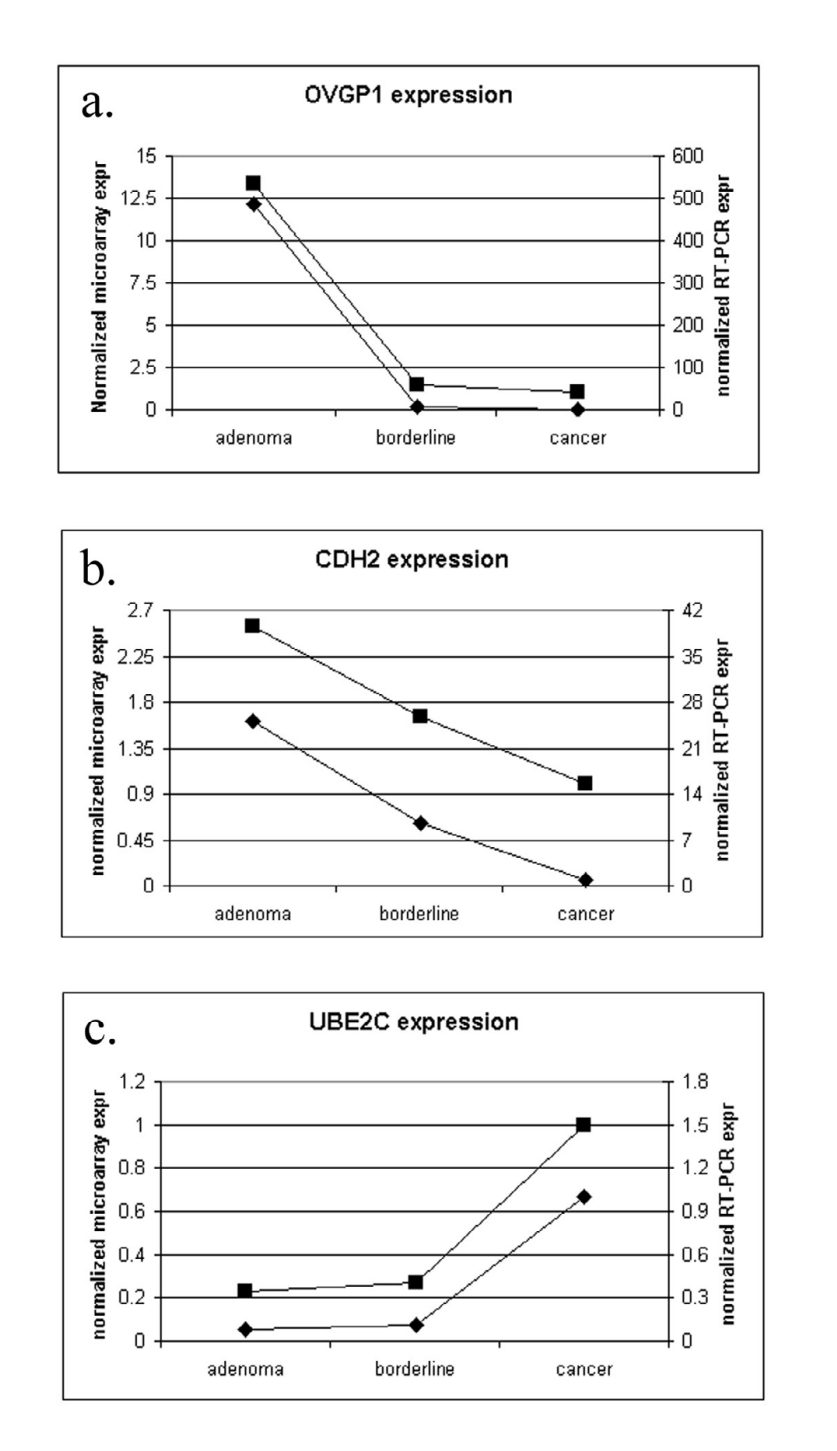
**RT-PCR validation of microarray results. **OVGP1 expression (a), CDH2 expression (b) and UBE2C expression as measured by RT-PCR (◆) and microarray (■). Patterns of change in expression were the same for each method.

To independently test the validity of the differential expression patterns determined by microarray, we measured the expression patterns of 3 representative genes in 6 tissue samples using quantitative real time RT-PCR [15]. Genes were selected from the microarray data set to represent a spectrum of statistical significance (Table 3). In all cases, the results of the quantitative RT-PCR analyses confirmed the differences detected in the microarray studies (Figure 4).

**Table 3:** Genes expression changes verified with quantitative RT-PCR.

**Gene Name**	**Gene Symbol**	**ANOVA rank**	**p-value**
ubiquitin-conjugating enzyme E2C	UBE2C	1	1.18E-7
cadherin 2, type 1, N-cadherin	CDH2	177	0.00042
oviductal glycoprotein 1, 120 kDa	OVGP1	739	0.0058

### Functionally related genes display correlated changes in expression between benign malignant tumours

**Figure 5:**
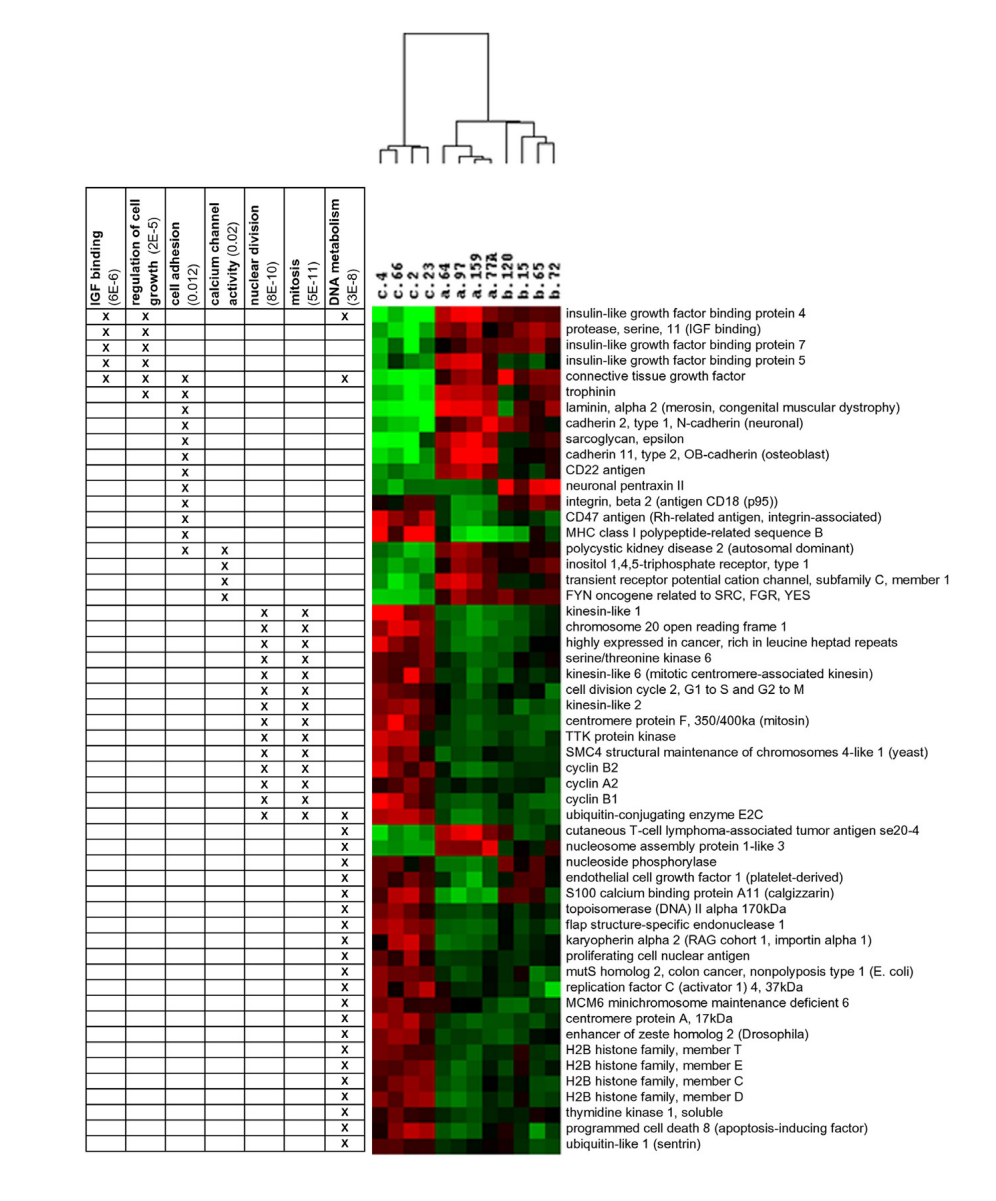
**Over-represented functional categories of differentially expressed genes. **Differentially expressed genes with low expression in cancer were able to bind insulin-like growth factor (p < 6 × 10^-6^) or functioned in cell adhesion (p < 0.012) and calcium channel activity (p < 0.02). Genes with high expression in cancer functioned in nuclear division (p < 8 × 10^-8^), mitosis (p < 5 × 10^-5^), and DNA metabolism (p < 3 × 10^-8^).

Two expression subgroups were evident in the list of 163 differentially expressed genes (Figure 3a): genes with low expression in cancer (first 61 genes of colour plot) and genes with high expression in cancer (last 102 genes of colour plot). To examine the possibility that these subgroups also correlate with differential gene function, we applied two functional profiling programs, EASE [16] and Onto Express [17]. Searching the gene ontology assignments for all genes in a list, these programs identify and assign statistical significance to the over-represented gene functional categories identifying common biological processes, molecular functions, cellular and chromosomal locations shared by genes in a list. Functional profiling revealed that the expression subgroups exhibited distinctly different gene functions (Figure 5). Genes in the expression subgroup with high expression in cancer were intracellular whereas the genes in the low expression subgroup were extracellular. Genes whose gene products function during cell proliferation and DNA metabolism dominate the high expression subgroup. On the other hand, gene products involving insulin-like growth factor binding, regulation of cell growth, cell-cell adhesion, and calcium transport activity were associated with the low expression subgroup.

## Discussion

### Microarray profiles of ovarian tumours are of potential diagnostic and prognostic significance

Gene expression profiling via microarray technology has previously been shown to be an effective tool for the objective classification of established tumour types [18,19] and in some instances, for the identification of previously unrecognized tumour sub-types [20]. Applied to ovarian cancer, gene expression profiling has aided in distinguishing clear cell carcinomas [8,9], characterizing advanced stage ovarian cancer [5,6], and identifying genes differentially expressed between normal and cancerous ovarian tissue [4,7,10]. The experiments presented here were designed to elucidate gene expression changes in ovarian tumours of differing malignant potential. In many instances, genes that we identified as differentially expressed across malignant potential were previously determined to be differentially expressed between normal and cancerous ovarian tissue including ERBB3 [10], ubiquitin carrier protein[10], and E-cadherin [4]. We also correctly classified 92% of tumours from patients who did not receive chemotherapy prior to surgery into their proper histopathological group. These results are consistent with earlier findings and indicate that gene expression profiling can effectively distinguish between malignant and benign ovarian tumours.

One particularly promising result emerging from our study is that expression profiling may be useful in predicting recurrence in patients treated with chemotherapy prior to surgery. We find that the microarray patterns of ovarian adenocarcinomas obtained from patients treated with chemotherapy prior to surgery clustered either with the benign tumours or with the malignant adenocarcinomas. Serum CA-125 levels indicate that patients whose samples clustered with the benign tumours have remained disease free for more than 3 years after surgery while those patients whose samples clustered with the malignant tumours recurred within 2 years of the initial treatment. Clearly, the testing of additional patient samples will be needed before definitive conclusions can be drawn. However, the preliminary results are consistent with the hypothesis that gene expression profiles of samples removed on the day of surgery may predict recurrence and would therefore be an indicator of the long-term effectiveness of chemotherapy administered to patients prior to surgery.

### Expression profiles indicate that borderline tumours are not a distinct disease

Our microarray data are, in general, most consistent with the hypothesis that borderline ovarian tumours represent an intermediate stage between the benign and malignant tumours. Borderline tumours of the ovary display many but not all characteristics of malignancy including nuclear atypia and increased mitotic count, usually in the absence of stromal invasion [21-24]. Whether the borderline tumour is a precursor to the fully malignant ovarian carcinoma or a disease distinct from invasive carcinomas is a topic that has been debated since the International Federation of Gynaecologic Oncology added the borderline tumour to the classification of ovarian tumours in 1972. Distinct disease states are expected to show discrete gene expression patterns when analysed by microarray [3]. Our analysis identified only 5 genes (Figure 3e) with increased expression distinctly correlated with borderline tumours. On the other hand, for 40 of the 163 genes displaying a significant change in expression between benign and malignant ovarian tumours, borderline tumours display an intermediate expression level (Figure 3c). In all other cases, (118 genes) borderline expression mimicked either the benign adenoma (102 genes) or malignant adenocarcinoma (16) tumours. Thus, for these genes, borderline tumours appear to be a transitional state between the benign and malignant state. The five genes identified as characteristic of borderline tumours (Figure 3e) may constitute a reliable marker of borderline tumours. Interestingly, two of these genes, AGR2 and NPTX2, are physically linked to one another, mapping to p21.3 on chromosome 7.

### Many genes displaying altered patterns of expression between benign and malignant ovarian tumours are genetically linked

Genes physically linked to one another shared changes in gene expression between tumour types. For those cases where linked genes displayed a significant reduction in expression in malignant vs. benign tumours (Table 4), at least three explanations are possible. Perhaps the most likely explanation is that the change is due to a small deletion in a chromosomal region encompassing the affected alleles. Such deletional events are believed to be at the basis of the "loss of allele" (LOA) phenomenon, which is known to be a relatively common event in tumour development [25-27]. Another possibility is that these co-ordinated reductions in gene expression are due to regional changes in chromatin structure resulting in the reduced access of transcription factors to genes. Such epigenetic changes are typically associated with the hypermethylation of so-called "CpG islands" in or around genes [28-30]. Indeed, it has been well documented that the silencing of many tumour suppresser genes and genes involved in DNA repair and apoptosis in cancer cells is the consequence of DNA hypermethylation [31,32]. The third possibility is that the coordinated reductions are the result of completely independent mutational events. However, the probability that such independent events would repeatedly occur at linked loci seems low.

**Table 4:** Co-ordinately expressed genes sharing chromosomal location

**Gene Symbol**	**Location**	**Expression in cancer**	**Function**
KIF2C	1p34.1	Up	Nuclear division/mitosis
CDC20	1p34.1	Up	Regulation of cell growth
PMSB2	1p34.2	Up	Protein Catabolism
UBE2C	20q13.12	Up	Nuclear division/mitosis
STK6	20q13.2-q13	Up	Signal Transduction
RGS19	20q13.3	Up	Nuclear division/mitosis
PDGFRA	4q11-q13	Down	Regulation of cell growth
IGFBP7	4q12	Down	Regulation of cell growth
HNRPDL	4q13-21	Down	RNA binding
FYN	6q21	Down	Calcium ion transport
LAMA2	6q22-q23	Down	Cell adhesion
CTGF	6q23.1	Down	Cell adhesion
FOXM1	12p13	Up	Transcriptional regulation
CCND2	12p13	Down	Nuclear division/mitosis
ERBB3	12p13	Up	Signal Transduction

We also observed a co-ordinated increase in gene expression of physically linked genes in the malignant samples in several cases (Table 4). These changes may have been due to regional duplication or amplification events. Examples of such events have been previously documented in cancer cells [33-35]. It is also possible that at least some of these co-ordinated increases in gene expression are the consequence of regional hypomethylation events resulting in a more open chromatin configuration and a consequent increase in transcription factor accessibility. Genes located in proximity to transposable element sequences may be more prone to such epigenetic events [36].

In a few instances genes that were physically linked displayed opposing changes in gene expression between benign and malignant tumours (Table 4). It is possible that these disparate changes were due to independent mutational events or, perhaps more likely, to a regional relaxation of chromatin structure that permitted increased access of both positive and negative transcription factors.

Our finding that a number of the genes displaying a significant difference in expression among malignant and non-malignant tumours indicates that some caution must be taken in the functional interpretation of microarray results. For example, significant changes in the expression of only one gene in a physically linked group may be of functional significance although correlated changes in gene expression may result from a regional effect.

### Malignant ovarian tumours display expression profiles consistent with previously established features of cancer cells

Two common features of malignant cancer cells are increased cell proliferation and loss of cell adhesion [37]. Consistent with these general features, we found that the majority of differentially expressed genes with high expression in the malignant tumours belonged to functional categories associated with DNA metabolism and cell proliferation. We also report here that insulin-like growth factor binding, cell adhesion and calcium ion transport were gene functional categories over-represented among the genes significantly under-expressed in ovarian cancer.

**Figure 6:**
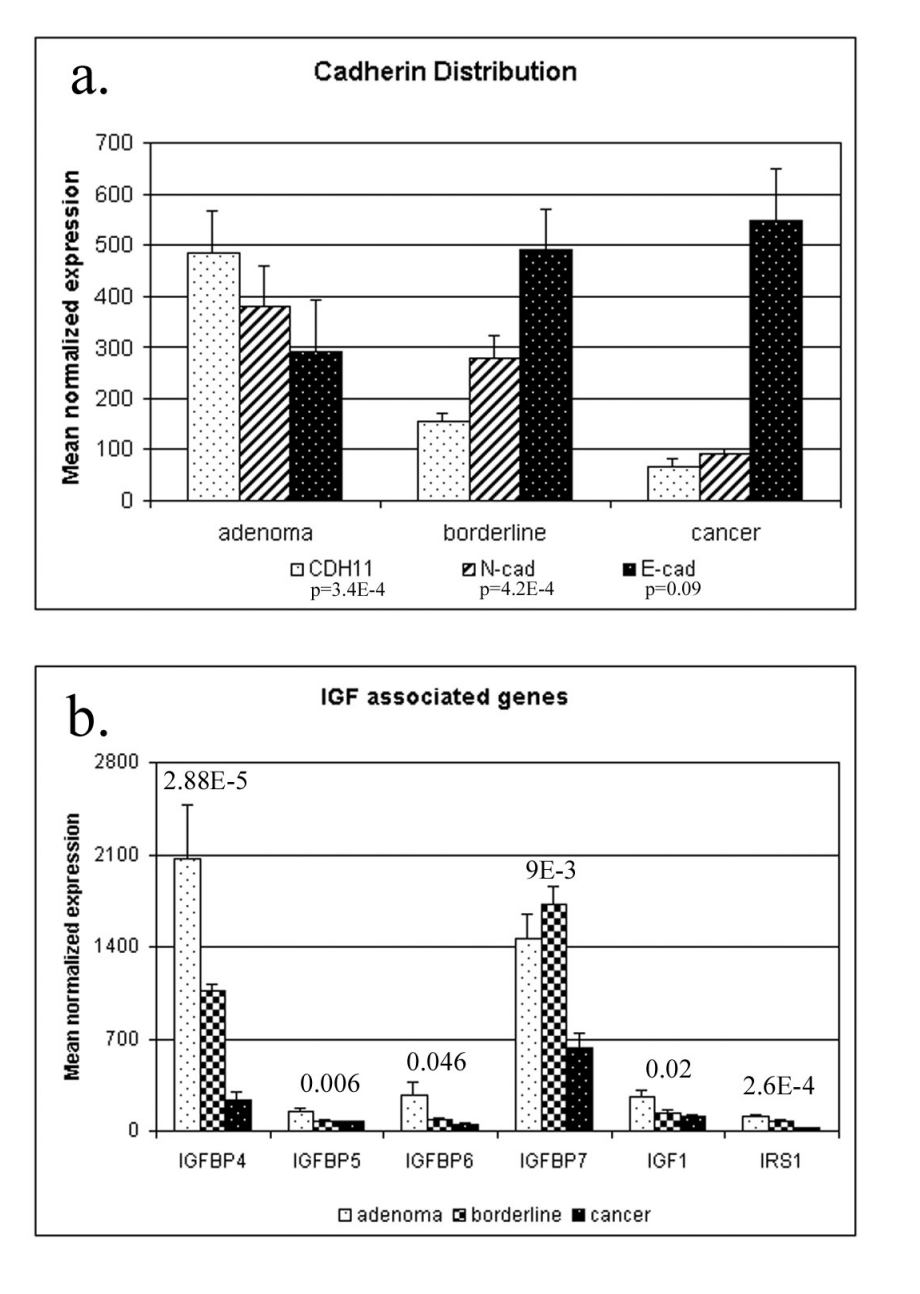
**Microarray expression of cadherins and insulin-like growth factor system genes. **E-cadherin expression contributes about equally to the cadherin distribution in benign adenomas but is the dominant component of the cadherin distribution in malignant adenocarcinomas (a). Insulin-like growth factor system components show lower expression in the adenocarcinomas relative to benign adenomas and borderline tumours (b). P-values associated with differential expression as analysed by ANOVA analysis are shown above each genes column graph.

The IGF system is a complex network of molecules involved in the normal growth and development of many cell types [38]. Disregulation of the IGF system through over-stimulation of the IGF1 receptor (IGF1R) has been implicated in tumour development and maintenance of the transformed phenotype [39,40]. The functional consequences of IGF1R over-stimulation include increased cell proliferation, cell survival and regulation of cell adhesion. The six specific IGF binding proteins (IGFBP-1 through -6) bind IGF in the serum and extracellular matrix, thereby reducing the bioavailability of IGF1 for receptor binding, as well as downstream signalling. Recently, elevated serum levels of IGFBP-2 at diagnosis were correlated with the likelihood of relapse, confirming the prognostic value of serum IGFBP-2 in choosing aggressive treatments for these patients[41]. Measuring serum levels of IGFBP-3 and IGF1 of healthy women proved useful in predicting a woman's risk of ovarian cancer [42]. Our analysis demonstrated significantly lower expression of IGFBP-4, -5, and -7 in the malignant adenocarcinomas than in the benign adenomas or borderline tumours (Figure 6b). IGFBP-2 and -3 were highly expressed but not differentially expressed across the tumour types. Furthermore, no IGF binding proteins appeared in the list of genes significantly up-regulated in cancer tissue. These findings suggest that loss of expression of IGFBPs in adenocarcinomas increases IGF signalling and its functional consequences, processes clearly associated with the clinical phenotypes of ovarian adenocarcinomas.

Over-expression of certain members of the IGF system increased sensitivity to IGF1 signaling in breast cancer cells [43] leading to increased cell proliferation. Insulin receptor substrate 1 (IRS1) is one member of the IGF system whose over-expression potentiated the effects of IGF1. Interestingly, IRS1 was significantly up-regulated in the benign and LMP tumours of our study (Figure 2). Considering the documented ability of the IGFBP's to reduce bioavailability of IGF1, increased expression of IGFBP's would be an appropriate cellular response to increased expression of IRS1.

Loss of cell adhesion molecules (CAM) is one mechanism proposed to induce the tissue invasion and metastatic capabilities acquired by cells during tumourigenesis[37]. Intra-abdominal spread of ovarian cancer via peritoneal implants is a hallmark of advanced stage ovarian cancer and can be linked to loss of cell-cell adhesion [44]. Our findings support the theory that loss of CAM in ovarian cancer is instrumental in cancer progression.

Cell-cell adhesion is often mediated through the cadherins, a family of transmembrane glycoproteins that require calcium to perform their adhesive functions. Well-documented changes in cadherin subtype expression correlate with the progression of breast and prostate cancer [45]. Recently, differences in the profile of cadherin subtypes expressed in normal and cancerous ovarian tissue were also shown to correlate with disease progression [46]. Support for cadherin switching in ovarian tumours is evident in our microarray data. Expression of N-cadherin (N-cad) and cadherin-11 (CDH11), the dominant subtypes in normal ovarian surface epithelium, were significantly higher in the benign and LMP tumours of our study than in the adenocarcinomas (Figure 4a). The intensity of change in expression between the benign adenomas and malignant adenocarcinomas for N-cad and CDH11 were 3.9 and 7.8 fold respectively, and both genes appeared in the list of top 163 differentially expressed genes. The LMP tumours in our study expressed N-cad and CDH11 at levels intermediate to adenomas and adenocarcinomas, suggesting an integral role for these cadherins in transformation to a malignant phenotype. Expression of E-cadherin, a major subtype seen in adenocarcinomas, increased approximately 2 fold from a benign tumour to either LMP or the adenocarcinomas (Figure 6a). This data documents the switch from a normal-like distribution of N-cad and CDH11 in the benign and LMP tumours to a cancerous profile dominated by E-cad expression.

Since cadherins are calcium-dependent cell adhesion molecules [44]and increased dietary intake of calcium correlates with a reduced risk of ovarian cancer [47], it is also interesting that calcium transport and calcium channel activity are gene functions that we found correlated with genes under-expresses in the adenocarcinomas. Thus it is also possible that altered functionality of the cadherins through changes in calcium availability, a parameter not measurable with microarray, may be involved in increasing a tumours' malignant potential.

## Conclusions

Our findings indicate that gene expression profiling can reliably distinguish between benign and malignant ovarian tumours. Expression profiles of samples from patients pre-treated with chemotherapy may be useful in predicting disease free survival and the likelihood of recurrence. Genes displaying co-ordinated changes in gene expression were often genetically linked suggesting that changes in expression for these genes are the consequence of regional duplications, deletions or epigenetic changes. Loss of expression of IGF binding proteins as well as specific cell adhesion molecules may be a significant mechanism of disease progression in ovarian cancer. A significant portion of the differentially expressed genes exhibited expression levels in borderline samples intermediate between benign adenomas and malignant adenocarcinomas, suggesting the borderline tumours are a transitional state between benign and malignant tumours.

## Methods

### Tumour Samples and RNA Isolation

A set of 18 primary ovarian tumours was obtained from the Ovarian Cancer Institute. This set of tumours was comprised of 4 benign cystadenofibromas, 4 carcinomas of low malignant potential (borderline carcinomas), 5 adenocarcinomas, and 5 adenocarcinomas from patients who received chemotherapy prior to surgery. This study was approved by the Institutional Review Board of the University of Georgia and of Northside Hospital (Atlanta), from which the samples were obtained Tissue was collected at the time of initial surgery and preserved in RNA Later (Ambion) within one minute of collection. For RNA isolation, each tissue (50 ± 25 mg) was homogenized on ice in 1.5 ml Trizol (Molecular Research Corporation) with a polytron homogenizer for about 30 seconds. RNA was isolated from the crude homogenate according to the manufacturer's protocols (Trizol, Molecular Research Corporation) with the following specifics. Linear polyacrylamide (5 μl) was added prior to homogenization to aid in RNA precipitation. Total RNA was further purified over an RNEasy (Qiagen) column using the manufacturer's cleanup protocol.

### Microarray Hybridization

Biotinylated target cRNA was generated according to the Affymetrix Technical Manual. In brief, 5–10 μg total RNA was converted to double stranded cDNA using Supercript II (Invitrogen). The cDNA was cleaned by phenol/chloroform extraction and ethanol precipitation. In vitro transcription of the cDNA with the High Yield RNA Transcript Labeling Kit (Enzo) yielded 50–100 μg of biotin labeled cRNA target. The cRNA was fragmented in a metal catalyzed acid hydrolysis to a length of 20–200 bp (by electrophoresis) and the fragmented cRNA was hybridized to the Affymetrix array (U95Av2) for 16 hours at 45C. Hybridized arrays were washed, stained and scanned according to the Affymetrix technical manual.

### Microarray Data Handling and Manipulation

Signal values were generated in two ways. Affymetrix signal values were generated from the .CEL file using the Affymetrix software MAS 5.0. The overall intensity of each array was scaled to an average intensity of 500. These normalized signal values were exported to Excel (Microsoft) for further analysis labeled the Affy-data set. Robust multi-array analysis (RMA) signal values were generated from the .CEL file using the espresso wrapper in the Affy library of the Bioconductor package in the R-statistical environment. The parameters of PM correction, background correction, normalization, and summary method were set to PM only, RMA, quantile, and median polish, respectively. The normalized signal values were exported to Excel for further analysis and will be referred to in this paper as the RMA-data set. For each data set, Affy-data set and RMA-data set, Pearson correlation coefficients were calculated (Microsoft, Excel) for a_97 vs. all other arrays. Higher correlation and lower standard deviation from the mean within groups was seen with the RMA data set, suggesting higher quality data.

### Clustering

Raw data output from the Affymetrics MicroArray reader is transformed into expression level values using the RMA method [48,49]of the "affy" package in the Bioconductor suite of the R statistical environment, and a text output file generated. This text file is then transformed into a *.gct file for input into the GeneCluster program. GeneCluster (Whitehead Insitute, ) was used to cluster the dataset on both samples and genes. Except where described below, default parameters were used. The SOM feature of GeneCluster was employed, and various values were explored for the "Cluster Range" and "Iterations" parameters. The 'Cluster Range' parameter sets the geometry of the clusterings that will be performed on the data. For instance, if 2–3 is entered, two cluster sets are produced. One has two clusters and the other three clusters. When entering a number, any set of factors of that number will create a clustering. If 9 is entered, a linear set of 9 clusters and a 3 × 3 matrix of clusters are produced. Marker analysis was also performed in GeneCluster, again using the default parameters.

### Quantitative RT-PCR

Total RNA (2 μg) from ovarian tissue was converted to cDNA using Superscript III (Invitrogen) primed with random hexamers under conditions described by the supplier. cDNA from this reaction was used directly in the Quantitative RT-PCR analysis. TaqMan probes and gene specific primers for three genes (RPL-29, UBE2C, OVGP1, and CDH2) were obtained from Applied Biosystems' Assay on Demand. The mRNA levels of the three genes were measured in 6 ovarian tumours and one normal ovary on the ABI Prism 7700 Sequence Detection System. PCR was performed using the TaqMan Universal PCR MasterMix (Applied Biosystems), according to the manufacturer's protocols with standard PCR cycling steps. Using RPL29 as a housekeeping gene and the normal human ovary RNA as a reference sample, the expression levels of UBE2C, OVGP1 and CDH2 were calculated according to the 2^-ΔΔCt ^method[15]. The Ct values of triplicate RT-PCR reactions were averaged for each gene in each cDNA sample. For each tissue sample assayed, the average Ct value for the gene of interest (UBE2C, CDH2 and OVGP1) was subtracted from the average Ct value of the housekeeping gene (RPL29) to obtain the ΔCt value. The ΔCt value of the reference sample was subtracted from that of the tumours to obtain the ΔΔCt value.

### Data Filtering and Statistical Analysis

The RMA normalised data set was analysed for probe sets likely to be absent in all samples. Probe sets whose maximum RMA normalised value across all samples was less than 5.2 were removed from further analysis. Analysing the remaining 10,520 probe sets, we applied an analysis of variance (ANOVA) to test the hypothesis that the mean expression values for all groups (adenoma, borderline and cancer) are equal. For each gene, the within group and between group variation was calculated and used to generate the F statistic and subsequent p-values [14]. Adjusted p-values were also calculated using Holm's method. The ranking of genes in order of significance was exactly the same for the un-adjusted and adjusted methods. However, the adjusted p-values were 1000 fold higher than the unadjusted p-values and only the top 50 genes were considered significant (p < 0.05). Since RT-PCR confirmed differential expression down to the 739^th ^statistically significant gene (see results section), we continued the analysis on the top 300 statistically significant genes. Genes whose groups means were identified as significantly different (p ≤ 0.001, 299 genes) in the unadjusted ANOVA were further analysed using Fisher's Least Significant Difference multiple comparison method. The differences between group means for all pairwise combinations of groups were calculated and compared to the least significant difference. Genes were declared differentially expressed if the pairwise difference between group means was greater than the least significant difference. Probe sets duplicated between pairwise comparisons and probes sets with a fold change value below 2.0 were removed, leaving 163 unique genes differentially expressed.

### Functional Profiling

Genes found to be differentially expressed in the statistical analysis were divided into two lists: genes overexpressed in cancer, and genes underexpressed in cancer. Each list was analyzed for over-represented functional categories based on molecular function, biological process, cellular component and chromosomal location using two different freeware programs: EASE [16]and OntoExpress [17]. Given a list of genes, EASE forms subgroups of genes based on the functional categories assigned to each gene. EASE assigns a significance level to the functional category based on the probability of seeing the number of subgroup genes within a category given the frequency of genes from that category appearing on the microarray. The 'EASE score' is the upper bound of the distribution of Jacknife Fisher exact probabilities. Onto Express identifies overrepresented gene functional categories in a manner similar to EASE and was used to verify results obtained from EASE. All information on chromosomal location was obtained from Onto Express since EASE does provide information on chromosomal location.

## Authors' contributions

SW performed microarray, basic statistical analysis and hierarchical clustering. SP performed SOM clustering and marker analysis. SD contributed to the statistical analysis. BB collected tissue and serum. EK directed SW and SP. JM contributed to the linkage and CA-125 analyses and directed SW.

## Supplementary Material

Additional File 1 The file complete_list.xls contains the gene name and chromosomal location for the 163 genes determined to be differentially expressed in this study. Click here for file
